# Investigating the Sustainability of Urological Surgery: A Retrospective Analysis

**DOI:** 10.7759/cureus.93904

**Published:** 2025-10-05

**Authors:** Rupert Vicary-Watts

**Affiliations:** 1 Vascular Surgery, Lancashire Teaching Hospitals NHS Foundation Trust, Preston, GBR; 2 Orthopaedics, Townsville University Hospital, Townsville, AUS

**Keywords:** healthcare education, healthcare sustainability, hospital sustainability, operating theatre, urology operation theatre, waste management

## Abstract

Climate change is the major health concern of the 21st century, and paradoxically, healthcare is one of the leading contributors to global warming. This study intends to investigate how urology can reduce its carbon footprint. A review of the literature highlighted how energy consumption - through air conditioning in particular - medical waste, and anaesthetic gases are the main causes of emissions from the theatre.

A retrospective analysis of the number of general anaesthetics (GAs) and anaesthetic gases used for all urological procedures over one month was conducted using electronic records. A questionnaire was provided to 30 theatre staff, assessing their insight into the carbon footprint of the department. It investigated waste management and engagement with the issue of the climate crisis. An analysis of the current waste streams and the amount of waste produced was also conducted.

Thirty-nine operations were done under GA, with sevoflurane used in 48% (n = 30) of all cases, and desflurane used in 3% (n = 7). Education and appropriate recycling facilities were identified as key areas within waste management that needed addressing. Clinical waste produced 850 tonnes per year at this Trust; domestic waste production was 725 tonnes per year; and recycling produced 160 tonnes of cardboard and 9 tonnes of plastic bottles. Discussion with the waste minimisation officer led to a planned introduction of a new waste stream into theatres. Poor staff knowledge on waste disposal and the new waste stream introduction was addressed with an in-person educational update on waste disposal. Second-cycle results showed a large improvement in staff scores prior to the new waste stream introduction.

This study identifies simple measures that can be introduced to greatly reduce the carbon footprint of urological operations. Energy consumption can be reduced with efficient theatre management, air conditioning awareness, and appropriate waste streams. Furthermore, the implementation of a well-structured educational programme, and readily available recycling bins, addresses high levels of inappropriate waste disposal.

## Introduction

Climate change is an apparent threat to human life, and the planet is in dire need of help. With freak weather patterns across the world, and a temperature rise of 2.1°C globally, homes, habitats, and lives are at risk of devastation [1]. Numerous health experts now consider climate change to be the most pertinent public health issue of the 21st century, and in 2015, 16% of all premature deaths were linked to pollution. There is a linear correlation between air pollution levels and cardiac disease, and food shortages are directly attributable to the rise in global temperatures, highlighting how climate change is directly impacting human health [2-5]. Therefore, it is disturbing to find that healthcare generates between 8% and 10% of global emissions. A sector that has a primary goal of improving and protecting life is, in fact, greatly contributing to something that is doing the opposite [6].

Three main perpetrators have been identified in the production of emissions within the theatre department: energy consumption, medical waste, and anaesthetic gases. Hospitals are large energy consumers, as they require high-powered medical equipment and ventilation systems to be running 24 hours a day. Medical waste has been increasing in volume due to higher demand for services, as well as a cultural shift towards disposable products. Lastly, anaesthetic agents act as greenhouse gases when they are released following their use for anaesthesia, which leads directly to global warming.

It is reported that the National Health Service (NHS) is responsible for 25% of public sector emissions [6]. Hence, it is unsurprising to find that the average energy consumption per square metre is nearly threefold that of a commercial building [7]. Furthermore, the operating theatre is vastly more energy consumptive than the rest of the hospital; theatres use up to six times the amount of energy, which is reported to be consumed at 7.15-12.61 GJ/m^2^ [6]. A vast amount of the energy demand in the operating theatre is due to the continuous use of heating, ventilation, and air conditioning (HVAC). HVAC needs to maintain the following criteria in order to ensure patient safety: achieve a comfortable indoor temperature, prevent the infiltration of air from outside the theatre, control the particle concentration and colony-forming units within the operating theatre, and produce the correct airflow to ensure sterility of the operating table. Due to these strict measures, 50%-99% of the energy consumed in theatres is attributed to HVAC use [6-8]. In many cases, theatres are in use 24/7; hence, the energy-intensive HVAC is also running continuously, leading to vast amounts of energy being consumed. The remainder of energy usage comes from energy-intensive surgical and anaesthetic equipment, high-powered lighting, and other standard electronics such as computers. 

Studies have investigated the reduction of HVAC usage across the hospital site overall; for example, Short and Al-Maiyah explored the use of natural ventilation and cooling for up to 70% of a hospital’s floor plan [9]. Furthermore, large cost savings of around $5,000 a year were predicted by Dyer and O’Mary with the introduction of occupancy sensors that switch the HVAC settings to a lower ‘unoccupied mode’ when the space is not being used for long periods of time [10]. However, due to the stringent nature of the criteria required for air conditions within theatres, not all techniques that are proposed can be implemented in the theatre department. Dyer and O’Mary’s suggestion poses difficulties for implementation within theatres, as even when unoccupied, certain air pressures are required to prevent the infiltration of outside air. Whilst the proposition of natural ventilation is simply not feasible for the theatre environment, it could lead to the introduction of contaminated or infectious air particles into the operating room.

Although the challenge of increasing the efficiency of HVAC systems in the operating theatre is more difficult, some strategies proposed for healthcare centres generically could be applied to the theatre department. For example, by replacing old, low-performing machines with new, efficient air conditioning units, and installing adjustable thermostats and zoning areas according to the air conditioning units, large energy savings of up to 1.8 kWh/m^2^ could be achieved [8]. Similarly, by performing diagnostics of the air handling units to assess their operating conditions, great energy savings could be made. A study showed that, often, some machines are working simultaneously to heat air and then cool it due to malfunctioning hardware [10]. Hence, by simply inspecting the units at regular intervals or updating to new technology, great energy savings can be made.

There is an estimated 181,000 kg of waste produced by UK healthcare each year, with the theatre department disproportionately contributing to 33% of this [11]. Over 75% of a hospital’s waste is analogous to domestic waste, and in the operating theatre, it is reported that as much as 90% of the waste that is disposed of is misclassified as hazardous when it would be suitable for other methods of processing [11]. This misclassification is costly both environmentally and financially. Clinical waste requires incineration at much higher temperatures, which produces large amounts of carbon dioxide (CO_2_). High-temperature incineration is reported to produce around 949 kg CO_2_ per tonne. There are a few reasons for misclassification, the first being issues over safety. When unsure of the appropriate waste stream to use, the safest place to dispose of an item is the clinical bin (unless it is cytotoxic waste). This, coupled with poor knowledge of the cost of waste treatment, and fear of being reprimanded for incorrect classification of waste as non-hazardous, has led to these high levels of unwarranted clinical waste.

Furthermore, surgical waste has been increasing in quantity over recent years. The rising population and increased strain on the health service have consequently led to higher levels of waste production. Moreover, the overriding need for maintenance of sterility does come at a cost, as packaging is found in multiple layers to prevent contamination. To accommodate sterility, along with other confounding demands such as cost and ease of use, there has been an adoption of single-use products over the last 30 years [12]. This transition, in conjunction with fears of infection control, has encouraged a ‘throw-away’ mentality within theatres which needs addressing. With the increasing amount of packaging and disposable products, recycling all appropriate waste would appear to be a suitable solution. However, a study from McGain et al. highlighted that the majority of potentially recyclable paper and cardboard was disposed of in waste streams that did not accommodate recycling [13].

Anaesthetic gases used for general anaesthesia contribute to climate change. These agents contribute to global warming, as they are known greenhouse gases that, due to their structure and composition, have a global warming potential over the next 100 years (GWP100) that is much greater than CO_2_, which has an equivalent GWP100 of 1. For example, sevoflurane has a GWP100 of 130, and desflurane has a GWP100 of 2,540 [14].

These anaesthetic agents are evaporated and mixed with medical gases, such as oxygen or medical air. The patient will then receive a stream of vapour through an airway device attached to a semi-closed system that allows for overflow and return of CO_2_. Less than 5% of the anaesthetic agents are metabolised by the patient, so all remaining gas is pumped out of the operating theatre by a scavenging system [15]. Once free within the atmosphere, the anaesthetic gases then act as greenhouse gases. They absorb and trap infrared radiation, consequently leading to rising global temperatures. To illustrate the global warming effect that anaesthetic gases have, Hanna and Bryson calculated the equivalent amount of CO_2_ that would be generated by a car journey when using an anaesthetic gas at an average flow rate for one hour. Hence, sevoflurane generates the corresponding amount of CO_2_ as an average-sized car over 6.5 km, nitrous oxide emits an equivalent amount as a 95 km trip, and desflurane produces an equal amount of CO_2_ as a 320 km journey [16,17]. 

A substantial contribution to the carbon emissions of a hospital is made by peri-operative care and the operating theatre, due to their wasteful and highly energy-intensive nature. Therefore, there is much that needs to be done to reduce the environmental impact of healthcare. This study will investigate the measures that can be put in place to effect change.

This article was previously presented at the 2023 World Urology & Nephrology Congress. 

## Materials and methods

Scope 1

**Figure 1:**
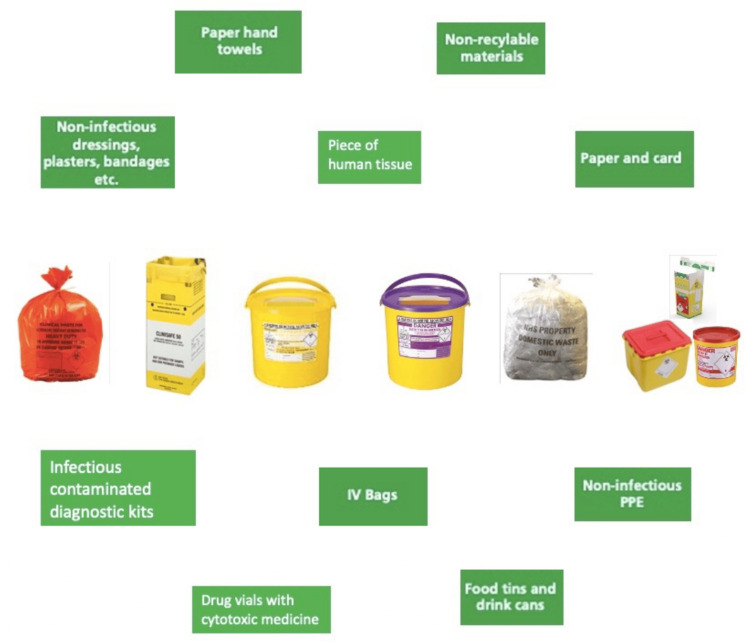
Waste management pictographic questionnaire

To investigate the issues surrounding waste management at the Trust, a pictographic question sheet was developed to assess the theatre staff’s knowledge of the waste management guidelines (Figure 1, see Appendix), to indicate whether waste was being correctly disposed of. To create the questionnaire, the Trust guidelines on waste management were used, in line with those set out by the Department of Health. The question sheet was provided to 28 staff on one day. The question sheet would identify areas of knowledge surrounding waste disposal that would require education. 

Data on waste tonnage was collected through an audit of invoices sent for waste processing, calculated by weighbridge assessment at the local waste facility. 

Following this, a new waste stream was rolled out for introduction, offering both environmental and financial benefits. To address the new waste stream and knowledge of waste disposal, an educational update was collated. Education was provided to theatre staff during audit days and drop-in training sessions in the department. It was provided by a single facilitator and focussed on being concise and informative, lasting 20 minutes. The educational update focussed on three main areas: (1) information on the impact of incorrect waste disposal, from an environmental and financial perspective; (2) differences between the old and new waste streams; and (3) confirmation of which specific items should be disposed of in each waste stream.

A revised question sheet was then provided to 33 staff before and after they had completed their waste management update over the course of two education audit days; therefore, the sessions were mandatory, with accompanying attendance monitoring. The question sheet was in the same pictographic format as the previous one, with only slight adaptations that improved on feedback from users of the first cycle.

To further tackle the points described above, appropriate signage was created that could be attached to walls in appropriate areas, such as the dirty utility or anaesthetic rooms.

Scope 2

To investigate the use of the anaesthetic agents that were being used, a retrospective analysis took place on all patients going through urology theatres over the period of a month, in December 2022. Data were collected on the type of gas used and at what flow rate it was administered during the procedure. This information was obtained from electronic records.

## Results

Scope 1

The initial question sheet identified a lack of knowledge of how to dispose of waste correctly. Upon looking at the results, no member of staff could appropriately place all waste items in the correct bin, with the largest percentage of staff scoring just over half on the quiz - getting five out of nine questions right (Table 1).

**Table 1:** Initial questionnaire scores The scores that staff recorded after answering the question sheet.

Question sheet score	Staff with score (% (n))
1/9	-
2/9	7 (2)
3/9	14 (4)
4/9	21 (6)
5/9	32 (10)
6/9	11 (3)
7/9	11 (3)
8/9	-
9/9	-

The distribution of staff scores attained on the repeat question sheet, following their educational update, is provided in Table 2.

**Table 2:** Final questionnaire scores The scores that staff recorded after answering the question sheet.

Question sheet score	Staff with score (% (n))
1/10	-
2/10	-
3/10	3 (1)
4/10	3 (1)
5/10	3 (1)
6/10	6 (2)
7/10	3 (1)
8/10	24 (8)
9/10	36 (12)
10/10	21 (7)

Scope 2

Of the total, 63 patients received operations in the urological theatre, and 39 received a general anaesthetic (GA). Two patients who received a GA had total intravenous anaesthesia (TIVA). Sevoflurane was used in 48% (n = 30) of cases, and desflurane was used in 3% (n = 7) of cases, as shown in Table 3. 

**Table 3:** Anaesthetic gas usage The number of cases for which the anaesthetic agents were used, and the average flow rate at which they were delivered.

Anaesthetic agent	Number of cases	Average flow rate (%)
Sevoflurane	30	2.2% ± 0.5%
Desflurane	7	3.6% ± 0.5%

## Discussion

Previous reports state that as much as 90% of waste from theatres is misclassified as infectious clinical waste when, in fact, it is non-hazardous and even recyclable [11]. When analysing the results from Cycle 1, our study somewhat supports these reports, as the scores attained on the question sheet were objectively low, suggesting that much of the waste produced in theatres is likely to be misclassified. The most common score that staff achieved was just over 50% (five points out of nine), and not a single member of staff was able to correctly place all waste items into the correct bin. When allotting an item of waste to a bin, the most common error was to place all items into the yellow clinical bin. With regards to safety, this is the choice of bin that staff fall back on when unsure of where to put a particular waste item. Although, unfortunately, clinical waste is processed with high-temperature incineration, which leads to the generation of large volumes of CO_2_ and processing fees that are much higher. Along with its transportation, the total amount of CO_2_ estimated to be produced by high-temperature incineration is 1,074 kg CO_2_ per tonne, and the Trust currently pays £850 per tonne for this type of treatment. By unnecessarily classifying waste as clinical due to poor knowledge of the waste policy, it has a large consequence on both the environment and the Trust.

However, the lack of knowledge of waste disposal can be addressed with an appropriate educational package. The results from Cycle 2 show a large improvement in scores for the waste question sheet. The distribution of scores in Cycle 2 was more negatively skewed, with 81% (n = 24) of staff scoring 80% or higher on the waste disposal questions. Hence, this study will hopefully have a positive influence by reducing the levels of waste that are misclassified, leading to savings in CO_2_ and Trust money.

This study is also set to have a further positive influence on the Trust's environmental impact, as, by spearheading the introduction of alternative treatment for clinical waste, vast quantities of CO_2_ will be saved yearly. When the switch from yellow clinical waste to orange hazardous waste takes place in the coming months, our carbon emissions for clinical waste treatment are estimated to be halved. Currently, waste is estimated to produce 1,074 kg CO_2_ per tonne at £850 per tonne, and the new stream will only produce an estimated 505 kg CO_2_ per tonne at £300 per tonne [18]. Stated on the intranet, the Trust at present generates approximately 850 tonnes of clinical waste per year. Hence, by using these figures, we can calculate an estimated saving of 483,650 kg CO_2_ per year and £467,500 per year, highlighting the huge benefits that will be experienced with the new initiative.

Of the patients who received a GA, 18% (n = 7) had desflurane as their anaesthetic agent. This demonstrates there is still work to be done to reduce the amount of desflurane that is used, due to its very high GWP100 [14]. The complete eradication of desflurane use may be more difficult, as it is argued by some authors to be superior to other agents in certain clinical situations [19,20]. Although the anaesthetic gases used in clinical practice have slowly changed over the last 70 years with the introduction of newer agents, there is hope that we can remove them from hospital formularies, as was seen by the Yale-New Haven health system in 2013 [14]. Other authors have taken a less abrupt stance and proposed that, when planning to use desflurane for an operation, a special request form should be required, hence putting a barrier between its unsolicited use by some practitioners and ensuring that it is only used when deemed clinically beneficial [21]. However, there is growing evidence for sevoflurane use, and research into its benefit over desflurane is building; therefore, by continuing along this trajectory, we can continue to phase out the use of desflurane [22,23].

To further reduce the greenhouse gas effect of anaesthetic gases, we can look to innovative technologies. Currently, all operating theatres are required to have gas scavenging technologies that prevent the build-up of dangerous levels of gases in the air. The waste gas is then vented out into the atmosphere. However, new technologies are being developed that recapture any waste gas, allowing it to be reprocessed [21]. Only 5% of anaesthetic gases are metabolised by the body; hence, with the continued growth of this recapture technology, a potential 95% of the anaesthetic gas we currently use could be reclaimed, with the prospect of then being reprocessed and reused [15].

TIVA is another technique that should be incorporated more readily in order to directly reduce the carbon footprint of operations. TIVA uses drugs such as propofol and other short-acting opioids to induce and maintain a GA, thus eliminating the need for anaesthetic gas completely. Research has investigated the environmental impact of TIVA and found it to have far less of an impact on the environment than any of the common anaesthetic gases that are used today [24]. Although the method requires additional plastic syringes and adjuncts, as well as an energy supply to run the electric syringe driver during the procedure, it was estimated to have the lowest carbon footprint when compared to the other anaesthetic gases. This suggests that anaesthetists could move away from anaesthetic gases completely, in pursuit of reducing their environmental impact.

As HVAC accounts for such a large proportion of the energy that is consumed by theatres, it is crucial that research explores ways that this can be reduced. Whilst the implementation of newer units and regular systems checks can help to increase the efficiency of our energy draw, newer techniques, such as non-operational modes, can actively cut the amount of energy required by theatres. Recent studies have found that the HVAC air inflow and outflow of non-operational theatres can be reduced by 30%-50%, whilst having no adverse effect on the air quality [25,26]. They concluded that noticeable energy savings were made during the process, whilst sterility was maintained in the operating theatre. Although these reports are promising, intensive investigation into this area is required in controlled environments to ensure the certainty of these results before any changes can be made to HVAC systems in operating theatres, where patient safety comes into contention.

The present study does have some limitations. Firstly, the sample size of staff who were requested to take part in the questionnaires may not be representative of the whole operating department. However, the staff who participated in the trial were across two departmental audit days, and, as such, you would hope that this is representative of the knowledge of the department as a whole. Similarly, when recording the anaesthetic gases used for the urological surgeries in the department, this was done retrospectively over a month's period, and, much of the time, anaesthetic gas use can be anaesthetist-dependent. As such, observing over a wider time period with more rotation of anaesthetists would help to provide better insight into the frequency of their use. Furthermore, anaesthetic gas use can vary depending on indication; as such, future studies should aim at collecting multi-specialty information across multiple sites for longer time periods. This would provide a better understanding of the current use and indication of certain pollutant anaesthetic gases. 

This study has reviewed and investigated the three main contributors to the carbon footprint of operating theatres: waste, anaesthetic gases, and energy consumption. It has implemented new strategies within the waste disposal process that are estimated to reduce the environmental impact of theatres and offer solutions to the greenhouse gas effect by highlighting key areas that require further investigation in order to reduce pollution and energy consumption.

## Conclusions

Waste management was improved with a dedicated educational programme that encompassed in-person training and the introduction of alternative waste streams. Switching from high-temperature incineration to alternative treatments is beneficial for the environment. Furthermore, the use of pollutant anaesthetic gases in urological surgery is reducing, but with further investigation, much can be done to reduce their impact on the environment.

## Appendices

Waste management pictographic questionnaire
